# Population Structure of Invasive *Streptococcus pneumoniae* in the Netherlands in the Pre-Vaccination Era Assessed by MLVA and Capsular Sequence Typing

**DOI:** 10.1371/journal.pone.0020390

**Published:** 2011-05-26

**Authors:** Karin E. M. Elberse, Ingrid van de Pol, Sandra Witteveen, Han G. J. van der Heide, Corrie S. Schot, Anita van Dijk, Arie van der Ende, Leo M. Schouls

**Affiliations:** 1 Laboratory for Infectious Diseases and Perinatal Screening, National Institute for Public Health and the Environment (RIVM), Bilthoven, The Netherlands; 2 Department of Medical Microbiology and the Netherlands Reference Laboratory for Bacterial Meningitis, Academic Medical Center, Amsterdam, The Netherlands; Naval Research Laboratory, United States of America

## Abstract

The introduction of nationwide pneumococcal vaccination may lead to serotype replacement and the emergence of new variants that have expanded their genetic repertoire through recombination. To monitor alterations in the pneumococcal population structure, we have developed and utilized Capsular Sequence Typing (CST) in addition to Multiple-Locus Variable number tandem repeat Analysis (MLVA).

To assess the serotype of each isolate CST was used. Based on the determination of the partial sequence of the capsular *wzh* gene, this method assigns a capsular type of an isolate within a single PCR reaction using multiple primersets. The genetic background of pneumococcal isolates was assessed by MLVA. MLVA and CST were used to create a snapshot of the Dutch pneumococcal population causing invasive disease before the introduction of the 7-valent pneumococcal conjugate vaccine in the Netherlands in 2006. A total of 1154 clinical isolates collected and serotyped by the Netherlands Reference Laboratory for Bacterial Meningitis were included in the snapshot. The CST was successful in discriminating most serotypes present in our collection. MLVA demonstrated that isolates belonging to some serotypes had a relatively high genetic diversity whilst other serotypes had a very homogeneous genetic background. MLVA and CST appear to be valuable tools to determine the population structure of pneumococcal isolates and are useful in monitoring the effects of pneumococcal vaccination.

## Introduction

*Streptococcus pneumoniae* is a major human pathogen causing considerable morbidity and mortality throughout the world. The pathogen carries a large number of virulence factors, but its polysaccharide capsule is still considered the most important virulence factor [1], [2]. The capsule provides resistance to phagocytosis and is therefore important for the survival of the bacteria at the infection site. Reactivity of the capsular polysaccharide with specific antisera is the basis of the classical serotyping technique. Currently, over 90 pneumococcal serotypes are recognized and approximately a quarter of these serotypes are responsible for the majority of cases of invasive pneumococcal disease [3], [4], [5], [6].

In the Netherlands, the 7-valent vaccine Prevenar® was introduced in 2006 in a 2–3–4 months vaccination scheme plus a booster at 11 months of age. The 7 serotypes in the vaccine account for approximately 60% of the serotypes responsible for invasive pneumococcal disease in the Netherlands [7]. Since vaccination against the pneumococcus is based on capsular polysaccharides, immunization will put selective pressure on the pneumococcal population. Important vaccine effects following immunization could be serotype replacement and capsule switch. Serotype replacement, the replacement of vaccine types by non-vaccine types, is already seen in the U.S.A., where after the introduction of the vaccine the incidence of invasive disease in children younger than 5 due to vaccine types declined. In the USA the overall incidence of invasive pneumococcal disease (IPD) decreased from 24.4 to 13.8 cases per 100,000 individuals. Among children aged <5 years, the IPD rate decreased from 98.7 cases per 100,000 individuals in 1998–1999 to 23.6 cases per 100,000 individuals in 2005. However, serotype replacement is occurring, predominantly by non-vaccine serotype 19A. IPD cases of serotype 19A increased about 3 fold to about 9 cases per 100,000 individuals [8], [9], [10]. Capsule switch is the ability to transfer capsule genes, by which the bacteria will change its serotype but will keep its genetic background [11]. In the U.S.A. capsule switch was already seen 3 years after the introduction of the 7-valent vaccine. The Active Bacterial Core (ABC) surveillance program of the CDC revealed an isolate with an MLST type associated with serotype 4, which was serotyped as 19A. MLST data as well as sequence of crossover regions and capsular loci of putative recombinants, recipient and donor revealed the probable capsular switch [12]. Changes in genotype and serotype may have considerable consequences for future vaccination strategies. To monitor alterations in the pneumococcal population, both serotyping and genotyping methods are required.

The gold standard for serotyping is the Quellung or Neufeld test [13], [14]. This method is time-consuming and the type, group and factor sera are expensive because they should be kept in-house for the identification of all serotypes. Some novel molecular ‘serotyping’ methods are described that are rapid and cost-effective. Brito et al. introduced a multiplex PCR scheme by which via multiple PCRs 9 serotypes could be identified [15]. Another serotype specific PCR for 51 serotypes was described by Kong et al. [16] and was later extended to 90 serotypes [17]. Furthermore, a widely used conventional assay that is continually updated uses a sequential series of multiplex PCR reactions for 40 serotypes or related sets of serotypes (www.cdc.gov/ncidod/biotech/strep/pcr.htm) [18], [19], [20]. In these methods several PCRs should be performed to assess the serotype.

Using both Capsular Sequence Typing (CST) and MLVA (companion paper, Elberse and Nunes et al.) we may be able to monitor changes in the pneumococcal population. The CST is a newly developed molecular method to genotype the capsular locus in order to assess the serotype. The primers used in the CST are based on the publicly available sequences of the capsular genes of the 90 known pneumococcal serotypes [21]. In this report we used the CST and MLVA to create a snapshot of the composition of the pneumococcal population causing invasive diseases in the Netherlands before the introduction of the 7-valent pneumococcal conjugate vaccine. The current snapshot will enable comparison with the post-vaccination population and may provide valuable insights on vaccine induced changes such as capsule switch and serotype replacement.

## Results

### Capsular Sequence Typing compared with classical serotyping

The pneumococcal population used for this study was collected in the pre-vaccination era in 2004–2005 and was representative for the total Dutch pneumococcal population causing invasive disease. Isolates belonging to serotype 14 and 7F were isolated most often, among other prevalent serotypes, such as serotypes 1, 4, and 9V (Table 1). All isolates were isolated from patients with invasive pneumococcal disease. Eleven percent of the isolates (n = 120) were recovered from cerebrospinal fluid (CSF) and the rest of the isolates from blood (n = 1034). In children aged <5 years 35% (n = 35) were isolated from CSF (Table 2). Serotype distribution within these children was slightly different compared with the serotype distribution in patients ≥5 years of age. Predominant serotypes among the children were serotypes 14, 7F, and 6B (Table 3).

**Table 1:** CT assignment per serotype included in the snapshot.

CST	Freq^1^	Serotype	Freq^2^	Mut.Major^3^	ds^4^	dn^5^	Mut.Bentley^6^	Discrepancies and other remarks
CT01-01	82	1	82	-			-	
CT03-01	47	3	47	-			1	
CT03-02	2	3	2	1	1		-	
CT03-03	11	3	11	1	1		2	
CT03-04	1	3	1	1		1	2	
CT04-01	86	4	86	-			-	
CT05-01	8	5	8	-			7	
CT06A-01	9	6A	9	-			2	
CT06A-03	14	6A	13	5	2	3	5	
		6B	1	5	2	3	5	Serotype 6B according to Quellung reaction
CT06A-04	2	6A	2	2	1	1	-	
CT06B-01	41	6B	34	-			2	
		6A	7	-			3	Serotype 6A according to Quellung reaction
CT06B-02	2	6B	2	27	17	10	29	BLAST: perfect match with serotype 6B (AF246897)^7^
CT06C-01	6	6C	6	-			ND	Serotype 6C according to PCR
CT07F-01	139	7F	139	-			-	
CT08-01	72	8	72	-			-	
CT08-02	1	8	1	1	1		1	
CT08-03	1	8	1	2		2	2	
CT08-05	1	8	1	1	1		1	
CT09N-01	20	9N	20	-			-	
CT09V-01	86	9V	86	-			1	
CT09V-02	1	9V	1	1		1	2	
CT10A-01	17	10A	17	-			-	
CT10A-02	1	10A	1	1		1	1	
CT11A-01	15	11A	15	-			-	
CT12F-01	18	12F	18	-			10	
CT12F-02	1	12F	1	2	1	1	8	
CT14-01	95	14	95	-			-	
CT14-02	80	14	80	1		1	1	
CT15A-01	3	15A	3	-			-	
CT15B-01	8	15B	8	-			62	
CT15C-01	2	15C	1	-			10	
		17F	1	-			1	Serotype 17F according to Quellung reaction, 1 mm with CT17F-01
CT15F-01	1	15B	1	-			11	BLAST:perfect match with serotype 15F (CR931666),64 mm with CT15B-01
CT16F-01	12	16F	12	-			-	
CT16F-02	1	16F	1	1		1	1	
CT17F-01	6	17F	6	-			-	
CT18A-01	1	18A	1	-			-	
CT18C-01	26	18C	24	-			-	
		18B	2	-			-	18B and 18C are indistinghuisable according to Bentley et al.
CT18C-02	1	18C	1	14	12	2	14	BLAST: best match (97% identity) with several serotypes
CT18C-03	1	18C	1	1		1	1	
CT18F-01	1	18C	1					BLAST: best match (97% identity) with several serotypes
CT19A-01	42	19A	42	-			-	
CT19F-01	24	19F	24	-			-	
CT19F-02	10	19F	10	1	1		1	
CT19F-03	1	19F	1	1	1		1	
CT19F-04	1	19F	1	4	3	1	4	
CT20-01	7	20	7	-			-	
CT21-02	1	21	1	-			-	
CT22A-01	3	22A	3	-			-	
CT22F-01	23	22F	23	-			-	
CT23A-01	9	23A	8	-			-	
		23F	1	-				Autoagglutinable
CT23B-01	2	23B	2	-			-	
CT23F-01	68	23F	67	-			-	
		15C	1	-			36	Autoagglutinable
CT23F-02	1	23F	1	1	1		1	
CT23F-03	1	23F	1	1		1	1	
CT23F-04	1	23F	1	31	21	10	31	BLAST: Best match (99% identity) with several serotypes
CT24F-01	7	24F	6	-			33	
		40	1	-			80	Serotype 40 according to Quellung reaction
CT25F-02	6	25F	4	-			1	
		38	2	-			1	25F and 38 are indistinghuisable according to Bentley et al.
CT28A-01	1	28A	1	-			-	
CT29-01	2	29	2	-			1	
CT31-01	3	31	3	-			-	
CT33F-01	6	33F	6	-			9	
CT33F-02	7	33F	7	-			-	
CT34-01	2	34	2	-			-	
CT35F-01	4	35F	4	-			-	

1 Frequency of the CT.

2 Frequency of the serotype.

3 Number of mutations with the major CT of the particular serotype.

4 Silent mutation.

5 Non-silent mutation.

6 Number of mutations with the *wzh* gene segment published by Bentley et al. [21].

7 Also perfect match with *wzh* sequences of 6B isolates AY359459, AY359455, AY359449.

**Table 2:** Characteristics of the patients that were infected with the isolates included in the snapshot.

	Blood	CSF	Total
Age of patients			
0–1 yr	30	22	52
1–4 yr	35	13	48
5–9 yr	9	2	11
10–14 yr	7	0	7
15–19 yr	10	1	11
20–29 yr	21	2	23
30–39 yr	56	3	59
40–49 yr	72	6	78
50–64 yr	203	35	238
65–79 yr	391	32	423
80+ yr	200	4	204
Gender of patients			
Female	472	60	532
Male	541	59	600
Unknown	21	1	22
Isolation year			
2004	492	69	561
2005	542	51	593
Total	1034	120	1154

**Table 3:** Serotype distribution of pneumococci causing invasive diesease in patients in the age categories <5 and ≥5 year.

	<5 yr	≥5 yr
Serotypes	n (%)	n (%)
14	26 (26)	149 (14)
7F	10 (10)	129 (12)
4	4 (4)	82 (8)
9V	6 (6)	80 (8)
1	3 (3)	79 (8)
8	2 (2)	73 (7)
23F	6 (6)	65 (6)
3	2 (2)	59 (6)
19A	7 (7)	35 (3)
19F	5 (5)	31 (3)
6B	12 (12)	25 (2)
18C	5 (5)	22 (2)
Other	12 (12)	225 (21)
Total	100	1054

In general CST and serotyping were in close agreement (Table 1). Among the 1154 isolates there were 42 distinct serotypes and 64 different capsular sequence types (CTs). For 25 (60%) of the serotypes only a single *wzh* sequence was found, representing 482 (42%) of all isolates. For the remaining of serotypes 2 or more *wzh* sequences per serotype were found. In most cases the variant *wzh* sequences within a particular serotype were closely related and only differed in a few base pairs. However, there were exceptions where the variant sequences differed in many residues from the other variants. This was the case for CT06B-02, CT18C-02 and CT23F-04, which differed 27, 14 and 31 nucleotides from the most frequently occurring variant, respectively. This resulted in 10, 2 and 10 non-silent amino acid changes, respectively. BLAST analysis revealed that no perfect match could be found for the CT18C-02 and CT23F-04 sequence with any of the published *wzh* sequences. Although there was close agreement between serotyping and CST, there were 9 instances comprising 18 isolates (1.6%) where there was a discrepancy between the phenotypic and genotypic assignment. In 2 instances isolates were autoagglutinable in the serotyping and this may resulted in incorrect serotype assignment. Apparently there is not enough specificity within the *wzh* sequences to distinguish serotypes 18B from 18C, 40 from 24F and 38 from 25F. Analysis of the *wzh* sequences published by Bentley et al. [21] corroborates this finding. There was a single isolate, serotyped as 17F, that carried the same *wzh* sequence as a 15C isolate and was therefore assigned CT15C-01. Despite repeated analysis in serotyping and CST this discrepancy remained. The *wzh* sequences of CT15C-01 and CT17F-01 only differed in a single base pair and the serotype assignment of these isolates remains uncertain. A single isolate serotyped as 15B carried a *wzh* sequence that differed in 64 positions from CT15B-01. BLAST analysis revealed a perfect match with the serotype 15F *wzh* sequence reported by Bentley et al. [21]. We therefore assigned CT15F-01 to this isolate. The discrepancy between serotyping and CST of a single isolate with serotype 40 and CT24F-01 remained after repeated analysis. Remarkably the sequence of the segment of the *wzh* gene of the serotype 24F and serotype 40 isolates differed from that of the 24F and 40 sequences published by Bentley et al. [21] in 33 and 80 residues, respectively.

### Capsular Sequence Typing of serogroup 6 isolates

The 74 serogroup 6 isolates were shown to contain 6 distinct CTs within the serogroup. Analysis of the 31 serotype 6A isolates yielded 4 different sequences in the targeted part of the *wzh* gene and 7 of these isolates had the same *wzh* sequence as a subset of the 6B isolates assigned CT06B-01. The 37 serotype 6B isolates yielded 3 distinct *wzh* sequences and one of these sequences appeared to be identical to those found in some of the 6A isolates (CT06A-03). There were 2 serotype 6B isolates that carried a *wzh* sequence which differed in 27 positions from CT06B-01 in the 506 base pair sequence used for CST. BLAST analysis of this *wzh* sequence against GenBank revealed a perfect match with 4 serotype 6B sequences confirming the correct assignment of CST. Based on the publicly available sequence we designed and used a 6C specific PCR to distinguish serotype 6C from serotype 6A [22]. Of the 74 isolates 6 (8%) yielded PCR products and were assigned serotype 6C. These isolates carried a *wzh* gene sequence that differed from that of all other serotype 6A and 6B isolates and was assigned CT06C-01. Remarkably, all 6C isolates were obtained from patients aged between 55 and 75 years of age. The CT06C-01 sequence only differed in a single base pair from CT06B-01 and 2 or 3 base pairs from the CT06A-variants. In contrast, the sequence of CT06B-02 was different in 28 base pairs from CT06C-01. We also used the serotype 6C specific PCR on isolates serotyped as 6B to distinguish serotype 6D, however, no PCR product was obtained in these isolates.

### Multiple-Locus Variable number tandem repeat Analysis

**Figure 1:**
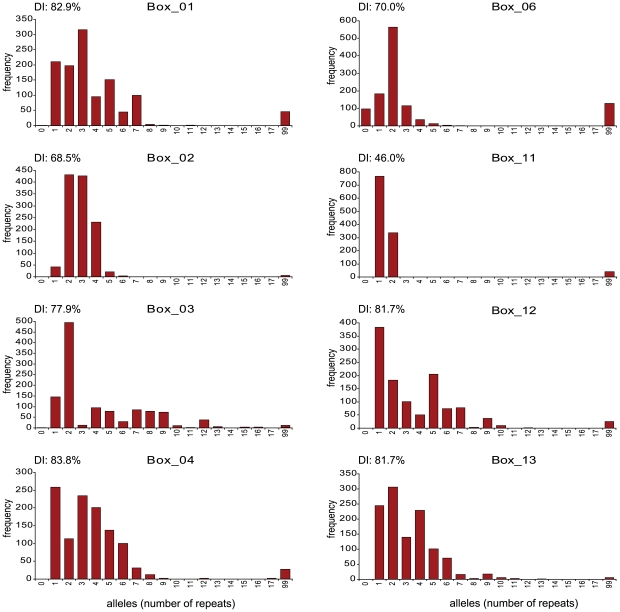
The frequency of the various alleles present in the 8 BOX loci used for MLVA. The graphs display the frequency of each allele (number of repeats) for each BOX locus. The Simpson's Diversity Index is indicated for each BOX locus. The number 99 was assigned if no PCR product could be amplified.

MLVA of the collection of 1154 pneumococcal isolates showed that the number of alleles and the degree of the variation in the number of repeats differed considerably among the BOX loci. Figure 1 shows the frequency of the alleles (number of repeats) for each BOX locus and the diversity indices (DI) per locus. BOX_04 was the locus with the highest degree of variation in the number of repeats (DI = 84%) while the BOX_11 locus carried either 1 or 2 repeats in the isolates resulting in a low DI of 46%. Overall the MLVA had a diversity index of 98%. The number of repeats varied between BOX loci, ranging from 0 to 17 for BOX_04 and from 1 to 2 for BOX_11. In 272 isolates (24%) one or more of the BOX loci could not be amplified even after repeated PCR. If BOX loci could not be amplified they were assigned allele number 99. Remarkably, BOX_06 could not be amplified in 128 (89%) of the 139 serotype 7F (CT07F-01) isolates accounting for 51% of all isolates in which one or more of the BOX loci could not be amplified. In 20 (29%) of the 68 serotype 23F (CT23F-01) isolates BOX_11 locus could not be amplified, which contributed another 6% to all isolates with an allele 99.

For the BOX_02 locus there were 82 isolates that yielded a PCR fragment of unusual size. Sequence analysis revealed a number of deletions in the region flanking the BoxB repeats in this BOX locus. In these cases the locus was assigned the number of BoxB repeats, irrespective of the mutations in the flanking regions. Remarkably, 66 (83%) of the isolates with CT14-02 had a mutation in the flanking regions. Also 10 isolates carrying a deletion in the flanking regions were CT06B-01, making up 25% of this CT in the collection. The BOX_02 locus was the only locus in which alleles with aberrant sizes were found.

### Snapshot of the pneumococcal population in the pre-vaccination era

**Figure 2:**
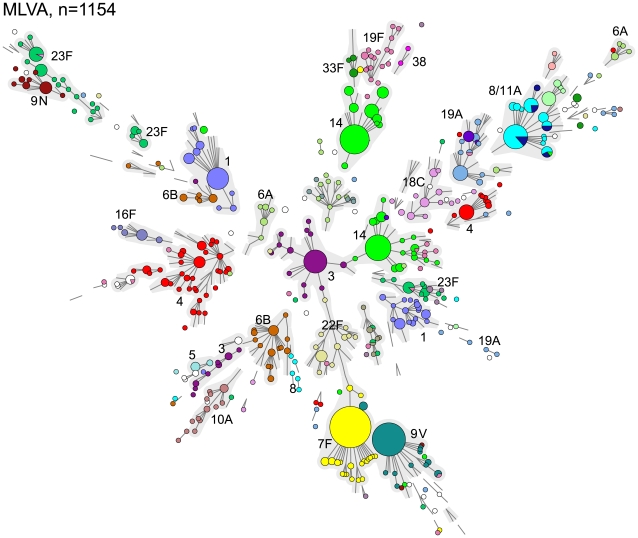
Minimum spanning tree of the results obtained by MLVA of 1154 pneumococcal isolates. The minimum spanning tree is based on the entire MLVA database (last accessed on October 20, 2010) with only the results from the current collection plotted within. The circles indicate the types and the size of the circles indicate the number of isolates. Lines linking two types denote a single locus difference between those types. The colors of the circles indicate the serotype of the isolates and complexes were indicated by grey halos.

**Figure 3:**
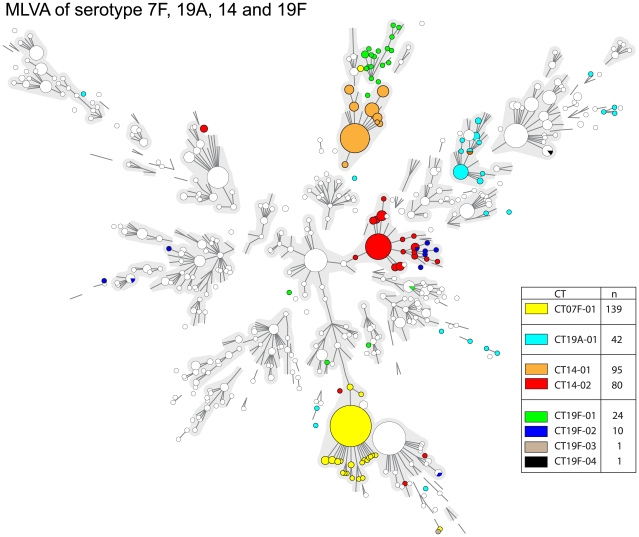
Minimum spanning trees of the results obtained by MLVA of 4 major serotypes. The minimum spanning tree is based on the entire MLVA database and the results of the pre-vaccination era collection (n = 1154) were plotted within in white nodes. The colored nodes designate the isolates of the 4 major serotypes described in the text. The colors of the nodes indicate the Capsular Type. For symbols and lines see Figure 2.

In Figure 2, a minimum spanning tree displays the 444 MLVA types that were obtained by MLVA of the 1154 isolates. In general, there was a strong correlation between MLVA type and serotype. However, several serotypes were distributed over various MLVA complexes. An example is serotype 14 which is divided into 2 distinct MLVA complexes. For some serotypes, e.g. 19F, the MLVA profiles were highly diverse. To illustrate the serotype dependent variation in MLVA the minimum spanning tree based on the MLVA of serotypes 7F, 14, 19A and 19F are depicted in Figure 3. Of the 139 serotype 7F isolates 108 (78%) had an identical MLVA profile and 136 (97%) isolates belonged to a single MLVA complex. Serotype 14 isolates clustered in 2 large complexes. Remarkably, the CST also yielded 2 CTs (CT14-01 and CT14-02) and the separation into the CTs was in full agreement with separation into 2 complexes obtained by MLVA. The distribution of the serotype 7F and 14 isolates over the MVLA complexes was similar irrespective of the age of the patients from which the strains were isolated. In contrast, more diversity was observed within the serotype 19A and 19F isolates. The MLVA of serotype 19F yielded many different MLVA types that were distributed over various complexes. Among the 36 serotype 19F isolates there were 35 different MLVA types. As a result virtually each isolate had its own MLVA type leading to a very high degree of diversity with a DI of 99.5%. However, the majority of the isolates belonging to CT19F-01 were closely related based on MLVA. Also, BOX_4 was absent in 12 of the 36 serotype 19F isolates. The collection contained 75 serotype 8 and 15 serotype 11A isolates. Although 21 different MLVA types were found, serotypes 8 and 11A were indistinguishable by MLVA. Inspection of the MLST profiles of serotypes 8 and 11A in the *S. pneumoniae* database (www.mlst.net) showed that the profiles of serotypes 8 and 11A are related but distinct. To verify this for our isolates, we performed MLST on 4 serotype 8 isolates and 4 serotype 11A isolates and this resulted also in related but distinct profiles.

### MLVA of serogroup 6 isolates

**Figure 4:**
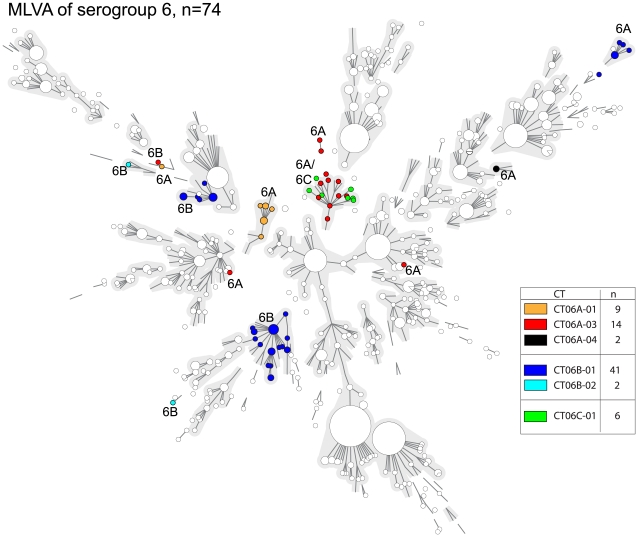
Minimum spanning trees of the results obtained by MLVA for serogroup 6. The minimum spanning tree is based on the entire MLVA database and the results of the pre-vaccination era collection (n = 1154) were plotted within in white nodes. The colored nodes designate the serogoup 6 isolates of this collection. The colors of the nodes indicate the Capsular Type and the serotype is indicated within the figure. For symbols and lines see Figure 2.

The serogroup 6 isolates possessed unusual characteristics both in CST and MLVA. Serogroup 6 yielded 3 serosubtypes, 6 distinct CTs and 53 MLVA types. Seven of the serotype 6A isolates had CT06B-01, a capsular sequence type that was also found in 34 serotype 6B isolates. In addition, there was a single serotype 6B isolate that carried a *wzh* gene sequence identical to that of 13 serotype 6A isolates (CT06A-03). The picture became even more complex when the MLVA data were used to construct a minimum spanning tree (Figure 4). With the exception of a single isolate all CT06A-01 isolates clustered in a single MLVA complex. The CT06A-03 isolates and the CT06C-01 isolates appeared to be closely related. The 6B isolates (CT06B-01) clustered in 3 MLVA complexes. The serotype 6A isolates that carried a *wzh* sequence that was indistinguishable from that of a subpopulation of serotype 6B isolates (CT06B-01) clustered as a separate MLVA complex which differed completely from all other serotype 6A and 6B isolates.

## Discussion

The aim of this study was to create a genotypic snapshot of the Dutch pneumococcal population in the pre-vaccination era of isolates isolated from patients with invasive disease. A newly designed molecular typing technique named Capsular Sequence Typing (CST) was used to assess the serotype. Isolates were also genotyped with MLVA to determine the genetic background from 1154 pneumococcal isolates.

Clustering of the MLVA profiles strongly correlated with serotype and CST distribution. The serotype 7F isolates had a diversity index of only 40% based on the MLVA profiles, indicating that the genetic background of this serotype is well conserved. Analysis of the MLST data of pneumococcal isolates from the UK present in the international publicly accessible MLST database (www.mlst.net) showed that serotype 7F isolates were highly clonal. The homogenous background of this serotype could suggest a rapid expansion or that this serotype evolves slowly. Serotype 14 was divided into 2 major MLVA types in our collection, and also in 2 major MLST types according to the UK data in the MLST database. For some serotypes there were many different MLVA profiles that often differed considerably in composition, reflecting a very heterogeneous genetic background. This was most pronounced for serotype 19F that yielded the highest diversity (DI = 99,5%) in the MLVA. Such result may suggest the 19F capsular gene cluster is transferred horizontally quite frequently among pneumococci with various genetic backgrounds. Although recombination of the capsular biosynthetic locus has previously been described [11], [12], [23], preferential horizontal transfer of a particular capsular gene clusters has not been reported. Also MLST revealed that serotype 19F isolates are highly diverse in the composition of the housekeeping genes. The publicly accessible MLST data do not represent the true distribution of STs in the UK and as such cannot be used to draw firm conclusions. However, the trend is obvious. Jefferies et al. created a snapshot for Scottish isolates genotyped by MLST [24]. Their analysis also resulted in highly clonal serotype 7F isolates and genetically diverse serotype 19F isolates. The number of isolates per serotype in their snapshot was only 15 isolates or less making comparison with our MLVA data somewhat inaccurate. However, from their study and from studies performed by others [25], [26], [27] it is clear that both MLST and MLVA yield type distribution that is strongly associated with serotypes. In the companion paper we compare the MLVA with MLST and PFGE on 263 isolates. Results of this study showed a very good congruence between MLST and MLVA.

CST is a molecular method to assess the serotype based on the *wzh* gene of the capsular locus. This gene was chosen for sequencing because it varies sufficiently between serotypes, but is conserved enough to amplify the same gene segment of the various serotypes using a single mix of primers. The *wzh* gene of the capsular locus is a regulatory gene important for the initial phosphorylation in the capsule production [28]. Alterations in the sequence of this gene may influence the level of capsule expression, but it is unlikely that it will affect the composition of the capsular polysaccharides. Further analysis of the level of expression of *wzh* variants of the same serotype may reveal differences in capsule expression. Such studies are important as increased expression of the capsule may require higher concentrations of antibodies to prevent pneumococcal disease. Vaccination may select for less sensitive variants and may even eventually lead to vaccine escapes. CST performed quite well and was able to confirm the serotype identified by the Quellung reaction in most cases. However, in some cases CST yielded ambiguous results where it did not match serotyping despite repeated analysis. Two discrepancies could be explained by an autoagglutinable character of those isolates. However, in 4 cases the discrepancy remained unexplained, additional typing based on other genes may be required to distinguish these serotypes. In 2 of these cases, the isolates with serotype 40 and 24F and isolates with serotype 18B and 18C, the MLVA profiles of the isolates were similar. In the other cases, the MLVA profiles were completely different. Evidently, the linkage between *wzh* sequence and serotype is not fully restricted. Horizontal transfer of complete capsular genes or parts thereof could change the composition of the genes or gene clusters, without changing the serotype. This may be particularly true for regulatory genes such as the *wzh* gene which may be involved in the level of production of the capsule, but do not determine the composition of the capsular polysaccharide.

The genotypic characteristics of isolates in serogroup 6 were remarkably diverse. Recent studies have shown that the serogroup 6 isolates are genetically diverse and two new serotype designated 6C and 6D have been identified [4]. However, from these studies there are indications that serogroup 6 may contain even more serotypes. Serotype 6B is associated with vaccine failure in the UK [29] and also in the Netherlands (unpublished data). It is believed that insufficient response to vaccination caused by the poor immunogenic character of the 6B polysaccharides may be responsible for the vaccine failures. However, the apparent heterogeneity in CST and MLVA within serotype 6B may suggest several other serotypes within serogroup 6. This may also play a role in these vaccine failure cases. Our MLVA data is supported by MLST of serotype 6A and 6C isolates that also showed overlapping genotypes [30].

In this study we designed CST as a molecular method to assess the serotype and used this method together with MLVA to create a snapshot of the composition of the Dutch pneumococcal population causing invasive disease in the pre-vaccination era. The use of both methods provides insights in the genetic background of the pneumococcus and the serotype specific capsular genes and can be used to observe changes in the pneumococcal population, including serotype replacement and capsule switch. Comparison with a snapshot made from the pneumococcal population after the introduction of the conjugate vaccine in the Netherlands may reveal such changes.

## Materials and Methods

### Isolates

Pneumococcal isolates were isolated from blood or CSF from patients with invasive pneumococcal disease and collected by the Netherlands National Reference Laboratory for Bacterial Meningitis (NRBM) in Amsterdam. The 1154 isolates used for the snapshot were isolated by 9 large Medical Microbiology laboratories referred to as the sentinel laboratories and represented approximately 25% of all cases from Dutch patients with invasive pneumococcal disease in the Netherlands in 2004 and 2005. In Table 2 the characteristics of the patients that were infected with the isolates used for the snapshot are shown. Serotyping was done at the Reference Laboratory using the Quellung reaction as previously described [13], [14]. For molecular analyses bacteria were grown in 1 ml Brain Heart Infusion Broth with 0.5% yeast-extract overnight at 37°C and 5% CO_2_. Of each culture 500 µl was heated for 10 min at 95°C and these lysates were either used directly or stored at −20°C until use.

The ethical committees (METC) of the University Medical Center Utrecht approved this study and waived the requirement for informed consent (METC Utrecht protocol number: 07-289/C), since cultures were obtained as part of the national surveillance program.

### Capsular Sequence Typing

CST primers targeting the *wzh* gene were designed based on the capsular biosynthesis gene sequences determined by Bentley et al. [21]. To be able to amplify the *wzh* genes of all pneumococcal serotypes in a single PCR, 3 forward and 4 reverse primers were designed and used as a mixture in the PCR (Table 4). Primer design was done using Kodon 3.5 software (Applied Maths, Sint-Martens-Latem, Belgium). The primers were designed with 5′-M13-tails to facilitate DNA sequencing with a single M13 primer set. To amplify the partial *wzh* gene 25 µl PCR mixtures containing Hotstartaq mix (Qiagen, Hilden, Germany), 10 µM of each primer and 5 µl 1∶10 diluted lysate were used. The PCR reaction was as follows: 15 min 95°C, 40 cycles of 20 sec 95°C, 30 sec 51°C and 30 sec 72°C followed by 7 min 72°C. PCR products were purified using ExosapIt (GE Healthcare Life Sciences, Chalfont St Giles, U.K.) according to manufacturer's protocol. One µl aliquots of the purified PCR products were used in sequence reactions with M13 forward and reverse primers using Big Dye Terminator technology (Applied Biosystems, Foster City, U.S.A.) on an AB 3730 genetic analyzer.

**Table 4:** Oligonucleotide primer sequences used in this study.

Assay	Primer	Primer seqeunce^1^	Coordinates	Accession nr
CST	CST_01-M13F	GTAAAACGACGGCCAGCATTCGCATATCGTTTTTG	3017	CR931635^2^
	CST_02-M13F	GTAAAACGACGGCCAGCATTCTCACATTATTTTTGATGT	11191	CR931710
	CST_03-M13F	GTAAAACGACGGCCAGCATTCGCACATCGTCTTTG	4520	CR931632
	CST_01-M13R	CAGGAAACAGCTATGACCTGAGCTCTTTTTTTCATGA	3547	CR931635^2^
	CST_02-M13R	CAGGAAACAGCTATGACGTGAACTCGTTTCTTCATGA	11719	CR931710
	CST_03-M13R	CAGGAAACAGCTATGACCCGAGCTCTCTTTTTCATAA	3185	CR931694
	CST_04-M13R	CAGGAAACAGCTATGACCCGAGCTCTCTTTTTCATGA	5043	CR931632
	M13F	GTAAAACGACGGCCAG		
	M13R	CAGGAAACAGCTATGAC		
Serotype 6C PCR	Sp-wciN-6C-F	TTTTACGCGCGATTAAAC	6724	EF538714
	Sp-wciN-6C-R	ACTAATACGACCAATCATCCC	7104	EF538714

1 Underlined sequences represent the M13-tail.

2 Sequences with accession number CR931632 to CR931722 were used for primer design.

All *wzh* variant sequences are made publicly available through www.MLVA.net.

### PCR for detection of serotype 6C

Detection of serotype 6C was performed with a specific PCR using primers targeting the *wciN* gene (Table 4). The primers were designed based on the publicly available sequence as established by Park et al. [22]. To amplify the *wciN* gene, 25 µl PCR mixtures contained Hotstartaq mix (Qiagen, Hilden, Germany), 10 µM of the forward primer and 10 µM of reverse primer and 5 µl 1∶10 diluted lysate. The PCR reaction was as follows: 15 min 95°C, 40 cycles of 20 sec 95°C, 50 sec 46°C and 30 sec 72°C followed by 7 min 72°C. The yield of a PCR product was assessed by electrophoresis on a 1% agarose gel and isolates were assigned serotype 6C if PCR products were obtained. Based on the available sequence of serotype 6D, this PCR would also yield a PCR product on serotype 6D isolates [6].

### MLVA

MLVA was performed as described in detail by Elberse and Nunes et al. (companion paper). Briefly, 8 VNTR loci were amplified in 2 multiplex PCRs in which one of the primers of each primer pair carried a distinct fluorescent label. The PCR products were mixed with fluorescently labeled size standard and sized on an automated sequencer. Assessment of the number of repeats in each locus was done using the GeneMarker software (Softgenetics, State College, Pennsylvania, USA). The resulting table with numerical profiles was imported into Bionumerics (Applied Maths, Sint-Martens-Latem, Belgium) for cluster analysis.

### Data Analysis

Data analysis and clustering were performed using Bionumerics version 6.1 (Applied Maths, Sint-Martens-Latem, Belgium). All sequences of the CST were assembled, edited, trimmed and assigned a CT. The CT is a composite assignment; the first part of the assignment is based on the phenotype assessed by conventional serotyping and the second part of the assignment is the consecutive number of the capsular type belonging to the same serotype. As an example, CT09V-01 designated the first variant in *wzh* sequence of an isolate serotyped as 9V.

Tables with the MLVA profiles were imported from the Genemarker software into Bionumerics and the profiles were clustered using a categorical similarity coefficient and displayed in a minimum spanning tree. In a minimum spanning tree circles indicate the types. The size of the circle indicates the number of isolates with that particular MLVA profile. The circles are linked based on the number of loci that differ in the MLVA profile. The lines linking 2 types in the tree denotes a single locus difference between these types. For assignment of MLVA complexes, the entire in-house MLVA database (available at www.mlva.net) was interrogated (last accessed on October 20, 2010). MLVA complex assignment was based on a maximum distance of one locus between related types. The minimum number of MLVA types in a MLVA complex was set to 3 with a minimum of 9 entries per MLVA complex, resulting in on average 3 isolates within an MLVA type. Complexes are depicted as halos surrounding the types.

The genetic diversity in MLVA profiles of the isolates was calculated with Simpson's index of diversity [31], [32], [33].
